# Genome Editing of Veterinary Relevant Mycoplasmas Using a CRISPR-Cas Base Editor System

**DOI:** 10.1128/aem.00996-22

**Published:** 2022-08-24

**Authors:** Thomas Ipoutcha, Fabien Rideau, Geraldine Gourgues, Yonathan Arfi, Carole Lartigue, Alain Blanchard, Pascal Sirand-Pugnet

**Affiliations:** a Univ. Bordeaux, INRAE, UMR BFP, Villenave d’Ornon, France; INRS

**Keywords:** CRISPR-Cas9, mycoplasma, animal pathogens, minimal cell, genome editing

## Abstract

Mycoplasmas are minimal bacteria that infect humans, wildlife, and most economically relevant livestock species. Mycoplasma infections cause a large range of chronic inflammatory diseases, eventually leading to death in some animals. Due to the lack of efficient recombination and genome engineering tools for most species, the production of mutant strains for the identification of virulence factors and the development of improved vaccine strains is limited. Here, we demonstrate the adaptation of an efficient Cas9-Base Editor system to introduce targeted mutations into three major pathogenic species that span the phylogenetic diversity of these bacteria: the avian pathogen Mycoplasma gallisepticum and the two most important bovine mycoplasmas, Mycoplasma bovis and Mycoplasma mycoides subsp. *mycoides*. As a proof of concept, we successfully used an inducible SpdCas9-pmcDA1 cytosine deaminase system to disrupt several major virulence factors in these pathogens. Various induction times and inducer concentrations were evaluated to optimize editing efficiency. The optimized system was powerful enough to disrupt 54 of 55 insertion sequence transposases in a single experiment. Whole-genome sequencing of the edited strains showed that off-target mutations were limited, suggesting that most variations detected in the edited genomes are Cas9-independent. This effective, rapid, and easy-to-use genetic tool opens a new avenue for the study of these important animal pathogens and likely the entire class *Mollicutes*.

**IMPORTANCE** Mycoplasmas are minimal pathogenic bacteria that infect a wide range of hosts, including humans, livestock, and wild animals. Major pathogenic species cause acute to chronic infections involving still poorly characterized virulence factors. The lack of precise genome editing tools has hampered functional studies of many species, leaving multiple questions about the molecular basis of their pathogenicity unanswered. Here, we demonstrate the adaptation of a CRISPR-derived base editor for three major pathogenic species: Mycoplasma gallisepticum, Mycoplasma bovis, and Mycoplasma mycoides subsp*. mycoides*. Several virulence factors were successfully targeted, and we were able to edit up to 54 target sites in a single step. The availability of this efficient and easy-to-use genetic tool will greatly facilitate functional studies of these economically important bacteria.

## INTRODUCTION

Mycoplasmas are minimal pathogens that belong to the class *Mollicutes* (1). They are characterized by a streamlining evolution from a Gram-positive ancestor, marked by drastic genome reduction (2–4). This evolution has led to the loss of diverse cellular functions, including cell wall production, various metabolic pathways, and efficient recombination machinery (5). These minimal bacteria are found in a wide range of host species, including humans and livestock. Many mycoplasmas are etiological agents of diseases that considerably reduce animal production yields and inflate veterinary health care costs (3, 6, 7). Currently, three of the most prevalent and economically relevant species worldwide are Mycoplasma gallisepticum, Mycoplasma bovis, and Mycoplasma mycoides subsp. *mycoides* (*Mmm*). M. gallisepticum is an animal pathogen that is listed by the World Organization for Animal Health (OIE) and is responsible for respiratory disease in poultry farms worldwide (8–10). M. bovis is responsible for bovine respiratory disease (BRD) as well as mastitis and reproductive disease (11, 12). *Mmm* is a member of the “*M. mycoides* cluster”, which is a group of five significant ruminant pathogens. It is the causative agent of contagious bovine pleuropneumonia (CBPP), an OIE-listed disease that can take chronic, acute, or hyperacute forms. In the hyperacute form, CBPP clinical signs include pericardial effusion, fever, and death (13). The control strategies for mycoplasmoses vary geographically and include animal movement regulation and the use of antibiotics, vaccines, and ultimately culling. In the context of increasing antibiotic resistance (14) and the relative lack of effective vaccines, better knowledge of the molecular basis of virulence and of host-pathogen interactions is required to improve curative protocols and design more efficient prevention methods (13, 15). However, the limited number of efficient genome engineering tools for many mycoplasmas, including the three species listed above, restricts functional genomics approaches and hinders efforts toward the production of rationally designed vaccines. During the past decade, cutting-edge genome engineering methods have been developed, and these methods rely on the cloning of the mycoplasma genome in yeast before editing with various genetic tools and back transplantation into a recipient cell (16, 17). Although offering unmatched possibilities to investigate and redesign complete genomes, these in-yeast approaches can be difficult to adapt to other species. They are still restricted to a small number of mycoplasmas and have not been adapted for any of the three pathogens considered here. Indeed, efforts to adapt these synthetic biology approaches to *Mmm* have been unsuccessful thus far, though they are now available for all other members of the *M. mycoides* cluster (18, 19). Only replicative (*oriC*) plasmids and transposon-based mutagenesis are available for M. bovis, and *Mmm* (20, 21). For M. gallisepticum, in addition to *oriC* plasmids (22) and transposon mutagenesis, a first tool for the targeted homologous recombination (HR) of short genomic regions has recently been reported (23).

Since 2012, CRISPR-based genetic tools have revolutionized the field of genome engineering in eukaryotes and prokaryotes. The typical CRISPR-Cas9 tool is based on the Cas9 nuclease and is guided to specific loci by single guide RNAs (sgRNAs) (24). Following DNA cleavage, repair occurs through distinct cellular mechanisms, including nonhomologous end joining (NHEJ) and homology-directed recombination (HDR). However, many bacteria, including mycoplasmas, lack efficient NHEJ and HDR repair machineries. Therefore, the CRISPR-Cas9 tools can only be used as a counterselection method (25). Base-editor systems (BEs) have offered a means by which to overcome this problem. Indeed, BEs combine a catalytically inactivated form of Cas9 (dCas9) fused with a cytosine deaminase (CBE) or an adenosine deaminase (ABE) and a uracil glycosylase inhibitor (UGI). At the molecular level, the mechanism of action of a CBE occurs as follows (Fig. S1): (i) an R-loop is opened in the target site by the dCas9-sgRNA complex; (ii) the single-strand DNA is accessible to the fused deaminase within a specific editing window; and (iii) the CBE catalyzes the deamination of cytosine into uracil, which is recognized as thymine after replication. Meanwhile, ABE converts adenine into inosil, which is recognized as guanine after replication (26–28). Base excision repair on edited nucleotides is prevented by UGI, and this improves the global editing efficiency (29). Therefore, CBE and ABE induce C:G to T:A and A:T to G:C transitions, respectively, and could allow for the insertion of a stop codon into genes of interest (26, 30).

Here, we first demonstrate the functionality of a base editor system in M. gallisepticum using a transposon to introduce the CBE and sgRNA into the bacterial cells. After optimization, three virulence genes located at various positions on the M. gallisepticum chromosome were successfully disrupted, and individual mutants were isolated. Next, a replicative plasmid and a transposon were used as vectors to evaluate this CBE in M. bovis and *Mmm*, and we demonstrate the high efficiency of this system for single-gene targeting in ruminant pathogens. Finally, multitarget mutagenesis using a single CRISPR guide was proven by the successful disruption of 54 insertion sequences in *Mmm*.

(A previous version of this manuscript was deposited on bioRxiv https://doi.org/10.1101/2022.03.09.483585. The manuscript is made available under a CC-BY-NC-ND 4.0 International license.)

## RESULTS

### Design and construction of a base-editor genetic tool for mycoplasma.

**FIG 1:**
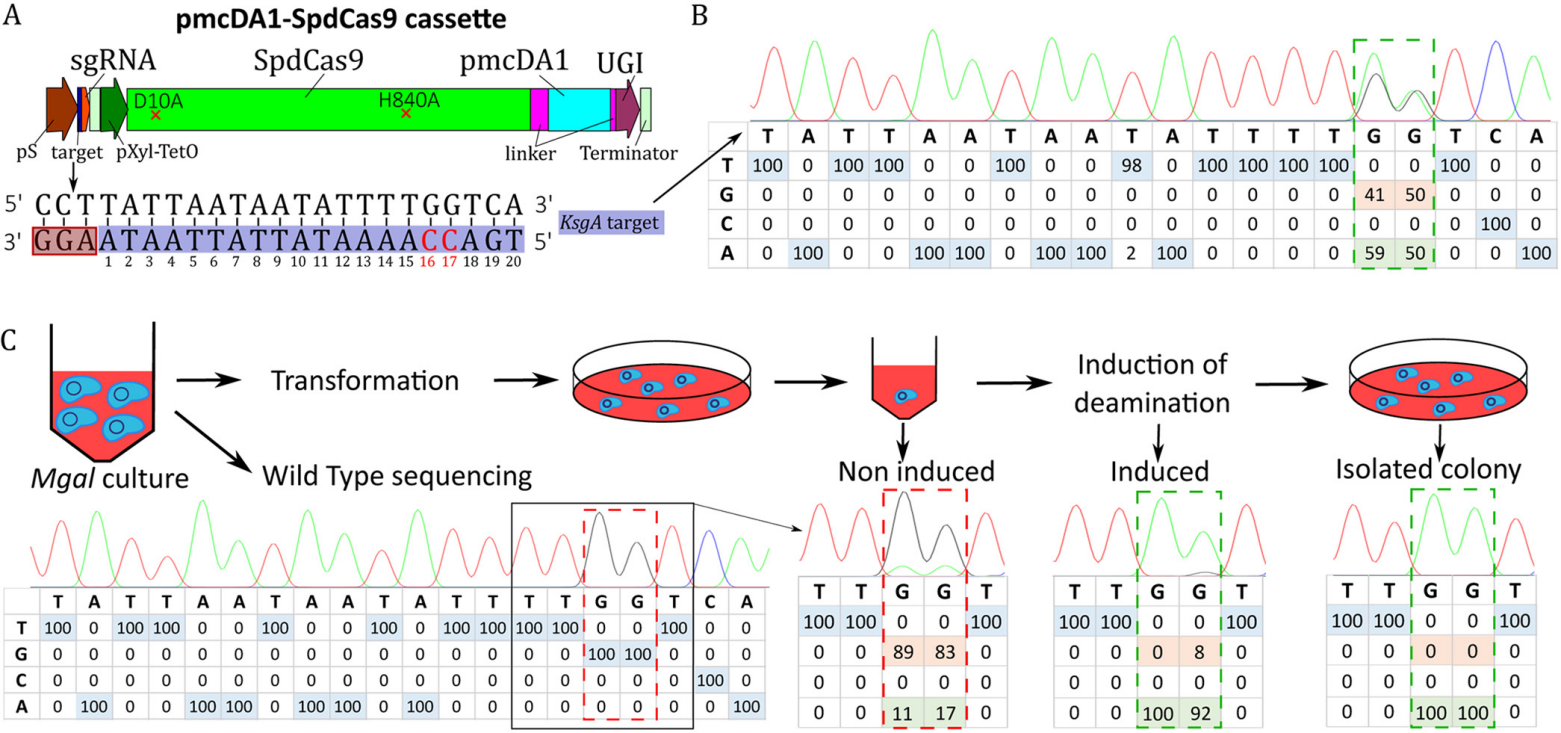
Targeting of the *ksgA* gene in M. gallisepticum using the pmcDA1 deaminase-encoding plasmid. (A) Scheme of the CBE expression cassette. The synthetic cassette is composed of (i) a sgRNA (red), including the 20-nucleotide target spacer (dark blue) under the control of the spiralin promoter (brown), and (ii) a codon-optimized hybrid protein composed of S. pyogenes inactivated Cas9 (SpdCas9, green), linkers (purple), the pmcDA1 deaminase protein (light blue), and uracil glycosylase inhibitor (UGI) (dark purple) under the control of the P*xyl/tetO2* inducible promoter (dark green). Fibril terminators from *S. citri* (light green) were added downstream of the sgRNA and hybrid protein expression cassettes. The sequence of the *ksgA* target is represented here. Cytosine residues that are susceptible to deamination are colored in red. The positions of bases in the target are indicated below each base, with numbering starting at the PAM sequence. (B). The percentage of bases found in the population of transformants was determined by Sanger sequencing of the complementary strand. The chromatograms were analyzed using EditR software and are represented in a table for each nucleotide position in the target sequence. Positions 16 and 17 are framed in a dotted rectangle. (C) Schematic diagram of the base-editing experiment in M. gallisepticum (*Mgal*) and an indication of various checkpoints (Sanger results and EditR analysis) for screening until isolated mutants are obtained.

Two different cytosine deaminases commonly used for base editing, rAPOBEC1 from Rattus norvegicus (26) and pmcDA1 from *Petromyzan marinus* (26, 27) (Fig. 1A, SI-1, SI-2), were evaluated as editing tools in mycoplasmas. The coding sequences of these two proteins were codon optimized, chemically synthesized, and fused to an inactive version of Cas9 (dCas9) from Streptococcus pyogenes (SpdCas9). All genetic elements were assembled within a Tn*4001*-derived transposon (31), resulting in plasmids pTI4.0_SpdCas9_pmcDA1 (Fig. S2B) and pTI4.0_ rAPOBEC1_SpdCas9 (Fig. S3A).

We selected the *ksgA* gene of M. gallisepticum as the first target to evaluate the ability of these recombinant CBEs to produce mutations in mycoplasma. The *ksgA* gene is nonessential and encodes an RNA methyltransferase that renders the bacteria sensitive to kasugamycin (32). A 20-nucleotide sequence (5‘-TGA**C_17_C_16_**AAAATATTATTAATA/AGG-3′), upstream of the SpdCas9 compatible protospacer-adjacent motif (PAM) sequence AGG (Fig. 1A and B), was chosen as the target for *ksgA* inactivation. On the basis of previous reports, the two cytosines at positions 16 and 17 upstream of the PAM sequence should be in the theoretical editing window of the two CBE systems (33). Deamination of C_17_ is expected to change a glutamine CAA codon into a TAA stop codon. After the transformation of M. gallisepticum with either pTI4.0_SpdCas9_pmcDA1 or pTI4.0_rAPOBEC1_SpdCas9 and an overnight induction of the inducible system with anhydrotetracycline (aTC) at 5 μg·mL^−1^ in growth medium (25), we analyzed the impact of the CBEs on cytosine deamination within the target site by PCR and Sanger sequencing on the global population of transformants (Fig. 1A and B; Fig. S3A). We observed only limited base editing for the two cytosines at positions 16 and 17 (2%) for the pTI4.0_rAPOBEC1_SpdCas9 transformants (Fig. S3), whereas base editing was more prevalent (59% of C to T conversion at C_16_, 50% at C_17_) for the pTI4.0_SpdCas9_pmcDA1 transformants (Fig. 1). There was no conversion in the control without the sgRNA. Given these results, the deaminase pmcDA1 appears functional in M. gallisepticum.

### Optimization of the CBE system and isolation of *ksgA* mutants in M. gallisepticum.

Given our initial results, the CBE system based on the pmcDA1 cytosine deaminase (pTI4.0_spdCas9_pmcDA1) was selected for further optimization. First, we determined the optimal inducer concentration by assessing the deamination level of targeted cytosines using the same experimental strategy and *ksgA* as the target after overnight induction with aTC at 0.1, 0.25, 0.5, 1, 2.5, or 5 μg·mL^−1^ (Fig. S4A). There was a notable leakage of expression in the absence of the inducer, with up to 25% conversion at position C_16_. In the presence of the inducer, the optimal concentration for base-editing was in the range of 0.1 to 0.5 μg·mL^−1^ aTC (>50% efficiency) (Fig. S4A). There was no detectable growth defect of M. gallisepticum with up to 1 μg·mL^−1^ aTC. However, the addition of aTC at 5 μg·mL^−1^ resulted in a marked reduction in growth, as shown by the almost complete absence of a pH shift in the broth medium after aTC induction (pH = 6.6 at induction versus 6.54, 5.41, and 5.3 after overnight induction with 5 μg.mL^−1^, 0.5 μg.mL^−1^, and no inducer, respectively). This result suggests that aTC is toxic for M. gallisepticum at high concentrations. The use of freshly prepared (<24 h) inducer was also necessary for maximally efficient base editing (data not shown).

Then, we studied the time of induction required for maximum efficiency in the CBE system. Inducer concentrations used for this experiment were 0.25 and 0.5 μg·mL^−1^, and base conversion was determined at two time points: after a 2 h induction or after an overnight induction (Fig. S4B). After 2 h, significant base conversion was already evident, relative to the noninduced condition, with up to 30% of base conversion at the two cytosine positions. However, overnight induction resulted in the highest efficiency, yielding a 2-fold higher conversion (approximately 60%) than that observed after 2 h of induction. Thus, the best induction conditions for the maximum efficiency of the mycoplasma CBE in M. gallisepticum were aTC at 0.1 to 0.5 μg·mL^−1^ and overnight incubation.

Finally, we performed a third experiment using the newly defined conditions to obtain isolated M. gallisepticum mutants using the pTI4.0_SpdCas9_pmcDA1 construct (Fig. 1C). Targeted *ksgA* sites were analyzed at four time points: before transformation (wild type), after transformation, after induction, and in isolated colonies selected on puromycin selective plates. In the noninduced condition, 11% of C_16_ and 17% of C_17_ were edited (Fig. 1C), indicating a leakage of the inducible promoter, as previously observed. After induction, deamination in the edited population was nearly complete, with 100% conversion of C_16_ and 92% conversion of C_17_ to thymine. Further screening of five isolated colonies confirmed that four carried mutations at the two targeted cytosines, whereas the last one showed an incomplete deamination profile, suggesting that deamination occurred during clonal expansion. Resistance to kasugamycin was confirmed by plating an isolated mutant on a Hayflick plate supplemented or not with the antibiotic (Fig. S5). Taken together, these results show that the pmcDA1-based CBE tool promotes base conversion and gene inactivation in M. gallisepticum with high efficiency.

### Determination of the editing window for maximum CBE efficiency in M. gallisepticum.

**FIG 2:**
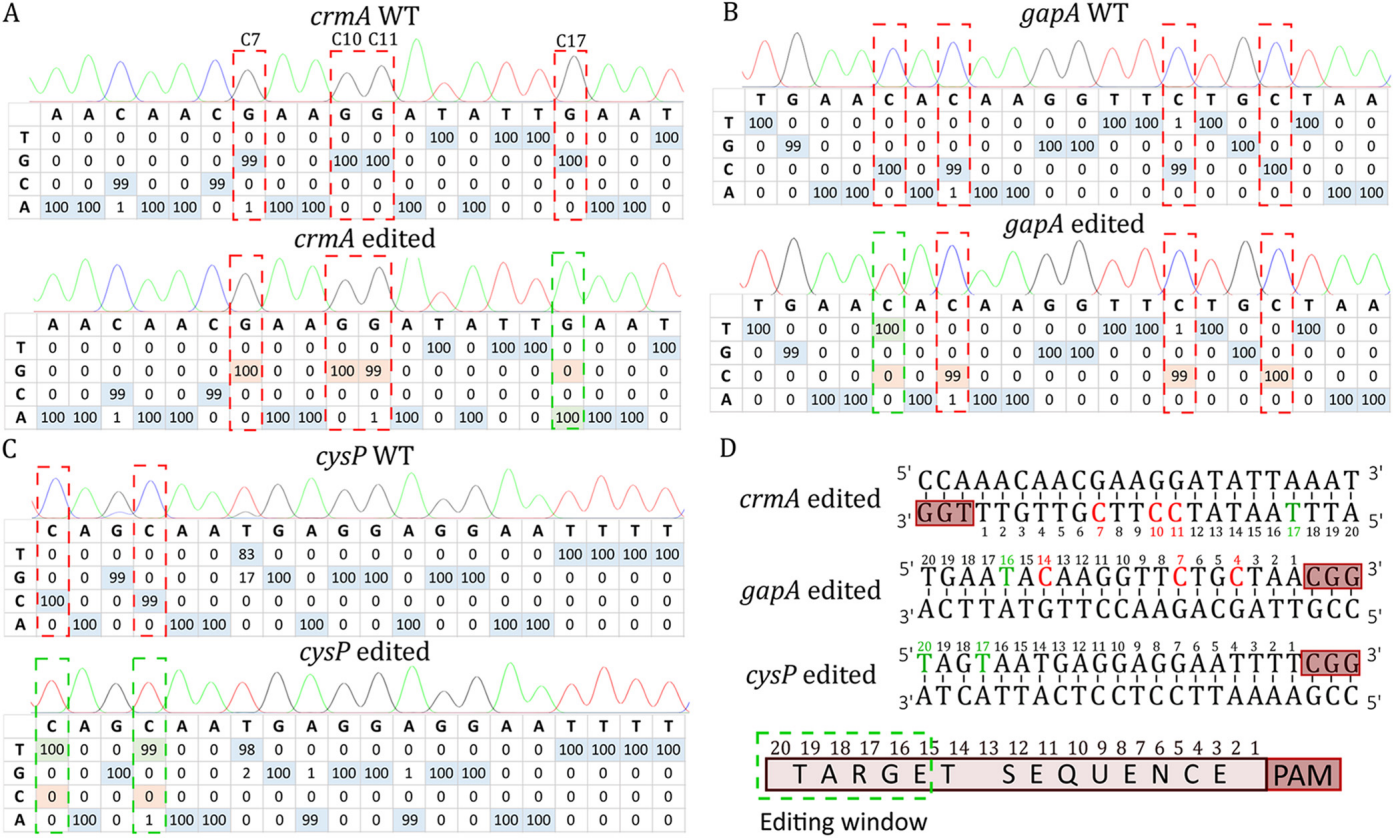
Targeting of three virulence factors of M. gallisepticum to explore the editing window of the CBE system in M. gallisepticum. The 20-nucleotide target sites were sequenced and are represented here for the *crmA* (A), *gapA* (B), and c*ysP* (C) genes before and after the base-editing experiments. Cytosines susceptible to deamination (or guanine in the reverse strand) are framed in red, and those that were deaminated are framed in green. The percentage of each base is shown in the tables, as determined using the Sanger sequencing.ab file and EditR software. Edited bases are highlighted in green. For the *crmA* target, the complementary strand was sequenced. (D) The 20-nucleotide target sites are shown for the three targets. The position of each base in the target is indicated below each nucleotide and corresponds to the nucleotide position in the target upstream of the PAM sequence. Red letters represent undeaminated cytosines, and green letters represent converted thymines. A scheme based on the three experiments and highlighting the editing window is shown at the bottom. As shown in the scheme, cytosines located at positions 16 to 20 can be CBE-targeted.

We investigated the base-editing window of our CBE system by targeting three other M. gallisepticum genes that encoded virulence factors: *crmA* (GCW_RS01080), *gapA* (GCW_RS01075), and *cysP* (GCW_RS01695) (Fig. 2). The *crmA* and *gapA* genes encode two primary cytoadherence proteins (34, 35). Meanwhile, the cysteine protease encoded by *cysP* has been shown to cleave chicken immunoglobulin G (36). In the *crmA* target (Fig. 2A) (5′-ATT**C**_17_AATAT**C**_11_**C**_10_TT**C**_7_GTTGTT/TGG-3′), cytosine residues C_7_, C_10_, C_11_, and C_17_ were potential deamination sites. After induction, C_17_ was the only base to be deaminated, with 57% conversion at the population level before the second plating (Fig. 1C). The screening of isolated colonies resulted in 5 of 10 clones showing a C_17_ to T_17_ transition (Fig. 2A, *crmA* edited). In the *gapA* target (Fig. 2B) (5′-TGAA**C**_16_A**C**_14_AAGGTT**C**_7_TG**C**_4_TAA/CGG-3′), C_4_, C_7_, C_14_, and C_16_ were potential deamination sites. After induction, C_16_ was the sole deaminated position, with 54% conversion estimated in the transformant population. In this population, 4 of 10 isolated clones showed a C_16_ to T_16_ mutation (Fig. 2B, *gapA* edited). Finally, in the *cysP* target (Fig. 2C) (5′-**C**_20_AG**C**_17_AATGAGGAGGAATTTT/CGG-3′), C_17_ and C_20_ were potential deamination sites. After induction and population analysis, the conversion levels were 76% and 77% for these two cytosines, respectively. All 10 of the 10 isolated clones showed mutations at the two positions (Fig. 2C, *cysP* edited). Thus, the editing window of the CBE system in M. gallisepticum ranged from positions 16 to 20, upstream of the PAM sequence (Fig. 2D).

### Application of the CBE system in M. bovis.

**FIG 3:**
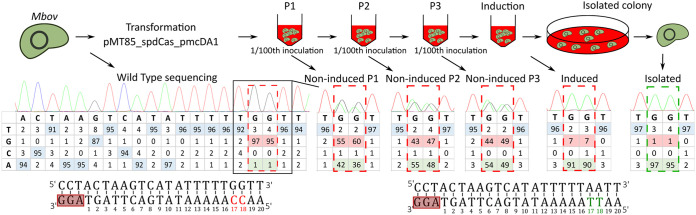
Disruption of the MnuA nuclease-encoding gene by base editing in M. bovis. A schematic diagram based on a base-editing experiment in M. bovis. After transformation of M. bovis (*Mbov*) with the plasmid pMT85_SpdCas9_pmcDA1, cells were propagated in liquid media supplemented with gentamicin. After three passages in liquid medium (P1 to P3), an inducer was added to the cell suspension. Aliquots were collected at each passage and after induction to monitor the target site sequence by Sanger sequencing and EditR analysis. Sequencing chromatograms of the complementary strand are presented along with the percentage of the base for each position of the target sequence. As shown in the diagram, cytosines susceptible to deamination are framed in red, and the edited bases are framed in green.

We initially evaluated the pTI4.0_spdCas9_pmcDA1, successfully used in M. gallisepticum, in M. bovis. However, we did not obtain M. bovis transformants using this CBE-carrying plasmid. This may be linked to the use of the *pac* resistance marker (puromycin), which has not been described in the literature for M. bovis. We therefore inserted the inducible CBE system into another transposon-based plasmid that carried a gentamicin resistance marker in order to generate pMT85_SpdCas9_pmcDA1 (Fig. S2). The resulting construct was evaluated in M. bovis by targeting the *mnuA* gene (MBOVPG45_0215) (Fig. 3). MnuA is a major membrane nuclease that has been suggested to play a key role in M. bovis virulence by degrading the neutrophil extracellular traps (NETs) produced by the host in response to the pathogen (37). In the target spacer (5′-AA**C**_18_**C**_17_AAAAATATGACTTAGT/AGG-3′), C_17_ and C_18_ stand as two potential deamination sites. The conversion of C_17_ would change a glutamine CAA codon into a TAA stop codon and disrupt the *mnuA* gene. M. bovis cells were transformed with pMT85_SpdCas9_pmcDA1 targeting the *mnuA* gene and the transformants grown in liquid gentamicin selective medium for three passages. Expression of the CBE system was induced overnight with aTC at 0.5 μg·mL^−1^, and M. bovis transformants were plated on selective medium to isolate colonies. Target sites were analyzed in the global population before transformation, after each passage following transformation (P1, P2, and P3) and after induction (for the cell suspension and isolated colonies) (Fig. 3). As previously found in M. gallisepticum, there was considerable leakage of the inducible promoter in the M. bovis population immediately after transformation, with ~50% of the cytosines being converted to thymines at positions C_17_ and C_18_ at P1, P2, and P3. Overnight induction with aTC increased the conversion level to 90%. The screening of three independent colonies showed that both cytosines (C_17_ and C_18_) in the *Mbov_mnuA* mutants had been converted to thymine. We then carried out a nuclease phenotypic assay to demonstrate that the mutations introduced into the *mnuA* gene led to its inactivation (38). Indeed, the tested *Mbov_mnuA* mutant was unable to hydrolyze DNA, whereas the wild type strain degraded it all (Fig. S6). These results demonstrate the portability of this CBE-based method to produce targeted mutations in M. bovis.

### Application of the CBE system in the Mycoplasma mycoides subsp*. mycoides* genome.

Next, we introduced the sgRNA and CBE expression cassettes into the replicative *oriC* plasmid pMYCO1 to demonstrate the flexibility of the mycoplasma CBE system. This plasmid is routinely used in several species of the *M. mycoides* cluster (21), including *Mmm*. The resulting pMYCO1_SpdCas9_pmcDA1 plasmid (Fig. S2) was used to target the nonessential gene *glpO* (5′-**C**_20_AA**C**_17_AA**C**_14_AATA**C**_9_GATAA**C**_3_AT/TGG-3′), which encodes the metabolic enzyme L-α-glycerophosphate oxidase, which is putatively involved in mycoplasma virulence (39). Indeed, GlpO catalyzes the oxidation of glycerol-3-phosphate, leading to the release of hydrogen peroxide (H_2_O_2_), a product known to contribute to cytopathic effects in host tissues. C_3_, C_9_, C_14_, C_17_, and C_20_ were potential deamination sites, with the conversion of the last three positions leading to three TAA stop codons. After the transformation of *Mmm*, we tested various inducer concentrations and induction times and monitored the conversion of the cytosines in the cell population (Fig. S7). Before the induction of CBE expression, the conversion of cytosines was observed as in M. gallisepticum and M. bovis, reaching up to 30% at position C_17_. The best results were obtained after overnight induction with aTC at 0.5 μg·mL^−1^. We observed the conversion for each cytosine within the target region and those located 14 nt and 22 nt upstream of the PAM sequence. The observed deamination at position C_14_ in *Mmm_glpO* had not been observed in *Mgal_gapA* (Fig. 2), suggesting an extended editing window. However, low conversion frequencies (20 to 30%) were observed at the editing window extremities (i.e., C_14_ and C_22_), whereas higher efficiencies were observed between C_17_ and C_20_ (60 to 70%). After subcloning on agar plates, the mutants showed diverse editing profiles at the four cytosine positions (mixed population and different fully deaminated cytosine combinations). Nevertheless, 2 of 10 isolated clones showed the expected four mutations (C_14_, C_17_, C_20_, and C_22_). A phenotypic assay to evaluate H_2_O_2_ production confirmed the inactivation of the *glpO* gene in these clones (Fig. S7E). Finally, after three passages in liquid medium without selective pressure and one passage in solid medium for isolation, we recovered plasmid-free *Mmm* mutants edited at *glpO*. These are the first reported site-specific mutants generated in *Mmm*.

### Inducing multiple mutations in Mycoplasma mycoides subsp. *mycoides* with a single CRISPR guide.

**FIG 4:**
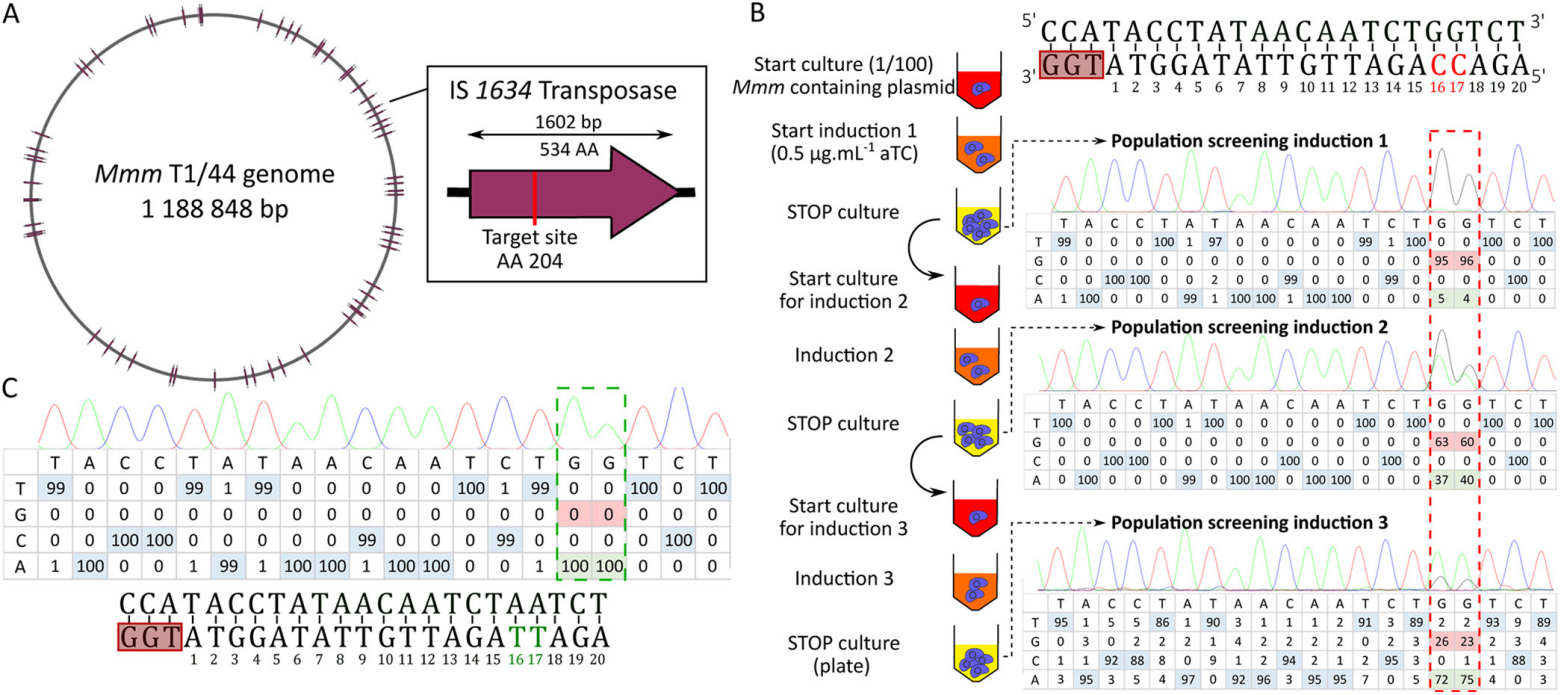
Multitargeting of IS*1634* copies in the *Mmm* genome. (A) Schematic diagram of the *Mmm* T1/44 genome with 58 complete or truncated copies of the IS*1634* transposases represented as solid lines. In the right panel, the targeted site within the IS*1634* transposase gene is represented as a red bar. (B) Schematic diagram of the three induction steps of the mycoplasma CBE system for targeting 55 IS*1634* sites using a single gRNA. The nucleotide sequence of the target is represented with the PAM sequence and framed by a red rectangle. Cytosines susceptible to deamination are shown in red. Fresh cultures of *Mmm* cells harboring the pMYCO1_SpdCas_pmcDA1 plasmid are represented as red-colored tubes. Cultures in exponential and stationary growth phases are indicated by orange-and yellow-colored tubes, respectively. Three consecutive culture steps were performed (including the starting culture) by a 1/100 dilution in fresh medium. At each step, induction was performed by adding aTC and incubating the cells until stationary-phase was reached. Deamination of target cytosines was evaluated by PCR and Sanger sequencing. The percentage of each base at each position was estimated from chromatograms using EditR software. The two positions of interest are highlighted in a dashed red rectangle. (C) Sanger sequencing results and base distribution in a selected isolated clone. Fully mutated positions are framed in green in the table and shown in green in the target sequence.

We tested the limits of the mycoplasma CBE system by targeting transposase-encoding genes that are associated with two families of insertion sequences, IS*1634* and IS*3*, present 58 and 26 times in the *Mmm* T1/44 genome, respectively (Fig. 4 and Fig. S8). As transposase gene sequences are highly conserved within each family, we attempted to target a maximum number of copies by using a single sgRNA per family. For the IS*1634* transposase, a 20-nucleotide spacer was designed to perform the inactivation of 55 of 58 copies (5′-AGA**C**_17_**C**_16_AGATTGTTATAGGTA/TGG-3′). Deamination of C_16_ on the reverse strand would change a glutamine CAG codon into a TAG stop codon at position 204 of the protein (Fig. 4A). For the IS*3* transposase, two 20-nucleotide spacers were designed for the sgRNAs, the first targeting the start codon of all 26 copies of the transposase (sgRNA1, 5′-**C**_20_ATATAAAAACCCCATTTCC/TGG-3′) and the second allowing the introduction of a stop codon in 22 of the 26 copies (sgRNA2, 5′-A**C**_19_AAGTGGAATACTATAAGT/TGG-3′) (Fig. S8). After transformation and induction, all target sites were assessed by PCR and Sanger sequencing (Fig. 4B and Fig. S8). We observed low base-editing efficiency for the *IS1634* target after the first induction, with only 5% conversion for C_16_ and 4% for C_17_. Two additional induction steps were carried out, resulting in an increase in base conversion of C_16_ and C_17_, yielding 37% and 40% after the second induction and 72% and 75% after the third induction, respectively. After the plating and PCR/sequencing screening of 20 isolated clones, two showed sequencing profiles that suggested that all IS*1634* sites had been mutated (Fig. 4C). Finally, full genome sequencing showed that 52 and 54 transposases out of 55 were inactivated in these two clones. For IS*3* inactivation, 66% and 77% conversion were observed in population analyses after three inductions using sgRNAs with spacers one and two, respectively (Fig. S8B, S8D). After the plating and subsequent PCR screening of 10 clones, one fully deaminated clone was obtained for each of the two sgRNAs (Fig. S8C, S8E). Full genome sequencing confirmed mutations at 22 out of 26 with sgRNA1 and 22 out of 22 target sites with sgRNA2 (Table S3). These results demonstrate the ability of the mycoplasma CBS system to edit numerous target sites in a single step.

### Evaluation of off-target mutations by whole-genome sequencing.

We investigated potential undesired mutations and evaluated the off-target activity of the CBE system in mycoplasmas by carrying out whole-genome sequencing on seven edited (mutant) clones, using both short-read (Illumina) and long-read (Oxford Nanopore Technologies) sequencing platforms: *Mgal_ksgA*, *Mbov_0215*, *Mmm_glpO*, *Mmm_IS1634_cl3.1.6*, *Mmm_IS1634_cl3.1.11*, *Mmm_IS3_cl4.1.2*, and *Mmm_IS3_cl5.1.18* (Table S3). We detected 5 to 36 undesired mutations in the sequenced genomes. Mutations were further analyzed and classified into three categories (Fig. S9A, Table S3): (i) sgRNA-guided off-target mutations possibly generated by the CBE system, if the targeted sequence was similar to the sgRNA spacer sequence and adjacent to an NGG PAM sequence; (ii) potentially unguided spurious deamination by pmcDA1 (30, 40) for all C to T or G to A mutations in genome regions with no similarity to the sgRNA spacer sequence; and (iii) other mutations that could not have been induced by the CBE system and that potentially occurred during passaging. Based on these criteria, 0 to 5 sgRNA-guided off-target mutations were predicted, representing 0% to 17% of the total mutations, with the exception of *Mmm_glpO*, for which 2 of 5 mutations were classified in this category. Two examples of off-target interactions were seen: the *ksgA* sgRNA targeting DNA in M. gallisepticum (mutation at position 1,313 in the genome) and the *glpO* sgRNA targeting DNA in *Mmm* (mutation at position 220,532 in the genome) are presented (Fig. S9B, S9C; Table S3). Comparing the undesired mutations distribution between the two independent *Mmm_IS1634* clones that were obtained with the same sgRNA and fully sequenced, we noticed that only 1 in 33 mutations was in common (and classified as an “other mutation”). This indicated that, at least in *Mmm* and with this sgRNA, the background mutations were not reproducible and changed from one clone to another. Such a distribution suggests that the vast majority of mutations were not caused by an sgRNA-driven off-target activity of the base editing tool. Spurious deamination represented 20% to 84% of mutations, whereas other mechanisms accounted for 11% to 50% of mutations. Interestingly, the number and percentage of spurious deamination that occurred when using the mycoplasma CBE system in *Mmm* were much higher when a three-induction protocol was used to maximize the efficiency of IS*1634* and IS*3* targeting (11 to 24 mutations) than when the single-induction protocol was used to target *glpO* (1 mutation). Thus, sgRNA-guided off-target mutations were relatively rare, whereas spurious deaminations accumulated with extended CBE activity.

## DISCUSSION

The functional genomics of *Mollicutes* has long been hampered by the absence of efficient genetic tools by which to generate targeted mutations. Here, we adapted a new genetic tool for *Mollicutes* that is based on CRISPR for direct mutagenesis. We evaluated its efficiency in three economically relevant animal pathogens.

CBE systems have proven to be highly efficient in diverse eukaryotic cells, including those of plants and humans (41, 42), as well as in prokaryotic organisms, including Klebsiella (43), Pseudomonas (44), *Clostridium* (45), *Streptomyces* (46), and *Agrobacterium* (47) species. Here, we designed and constructed two mycoplasma CBE systems, but our preliminary results suggested that only the pmcDA1-based CBE was active in M. gallisepticum (Fig. 1; Fig. S3). Both cytosine deaminases have been used successfully in bacteria (46, 48), but optimizing the expression levels of BE components, including sgRNAs, has been shown to be key in avoiding cell toxicity and in reaching a high rate of base conversion (49, 50). In this study, transformation experiments of M. gallisepticum with plasmids carrying the pmcDA1-based and rAPOBEC1-based CBEs did not suggest cell toxicity effects. However, the rAPOBEC1-based CBE was not efficient in M. gallisepticum, and further investigations are required to clarify why. In contrast, the pmcDA1-based CBE was highly efficient in generating point mutations in M. gallisepticum, M. bovis, and *Mmm* (Fig. 1 and 3; Fig. S7). Such contrasted efficiencies of rAPOBEC1 and pmcDA1-based CBEs have also been reported in other organisms (51).

Taking advantage of such a tool, we succeeded in targeting up to 54 IS*1634* targets in the *Mmm* chromosome with a single CRISPR guide. Multiple targeting of repeated sequences has been reported previously in eukaryotic and prokaryotic systems (48, 52, 53), but to our knowledge, this is the first time that so many target sites have been modified in bacteria, with the last record in E. coli targeting 41 loci (48). Noticeably, achieving such multiple targeting required tuning the protocol, including multiple rounds of induction of the base-editing system expression. In the best conditions, the global mutation efficacy in the bacterial population for a single target ranged from 50% to 100% (in *gapA* and *ksgA,* respectively) and led to the easy isolation of mutants. When targeting 54 sites, we observed a decrease of the global efficacy, with only 5% conversion in the bacterial population after the first induction. However, this efficacy reached 38% and 73% after a second and a third round of induction, respectively, and the screening of 10 to 20 clones was sufficient to successfully isolate clones with all or nearly all targets modified. The efficacy and specificity of CRISPR-Cas-based genetic tools rely on the ability of sgRNA to anneal with the target sequence. In our study, all of the sgRNAs we designed and evaluated in M. gallisepticum, M. bovis and *Mmm* gave comparable results, each time resulting in the expected mutation. More data may reveal some gRNA effect in terms of efficacy, but we have not observed such so far.

In order to evaluate potential off-target mutations induced by the mycoplasma CBE system, we selected seven mutants for whole-genome sequencing (Fig. S9, Table S3). An analysis of the detected mutations showed that only a few of them could have resulted from sgRNA-guided off-target deamination events. Spurious deamination of pmcDA1, which preferentially deaminates TC motifs (30), appeared to increase with extended induction periods, suggesting that the control of CBE expression could be crucial to reducing the frequency of undesired mutations. Such spurious deamination induced by CBE systems has already been reported in other bacteria, including Corynobacterium glutamicum and Bacillus subtilis, in which 9 and 19 SNVs, respectively, could be attributed to deaminase activity (54, 55). In the CBE system adapted here, the expression of the SpdCas9-pmcDA1-UGI hybrid protein is driven by the promoter P*xyl/tetO2*, which can be induced by aTC. Although we observed a clear increase in the frequency of deaminated bases in the three studied mycoplasmas after induction, the conversion process was already observed before induction (Fig. 1 and 3; Fig. S7). This indicates a certain level of promoter leakage in the three species, in accordance with another report on M. pneumoniae (56). Several strategies can be proposed to limit the background of undesired mutations, including reducing the induction time and using an improved inducible promoter, such as that recently developed for M. pneumoniae (56). In addition, the use of high-fidelity Cas9 variants or CBE variants (33) may also improve the specificity of the genetic tool. Finally, the elimination of the CBE system after mutagenesis is also crucial for avoiding the accumulation of undesired mutations over time. This can be achieved for the CBE systems based on the pMT85_2Res and pMYCO1 backbones. Indeed, all genetic elements flanked by the *res* sequences in the pMT85_2Res can be removed from the chromosome using dedicated resolvase activity (57), and the *oriC* plasmids can be lost after a few passages in nonselective medium (58). Alternatively, CBE constructs can be enhanced with a CRE-Lox system, which is functional in some mycoplasma species (23, 56, 59).

In the era of synthetic biology, with the fast expansion of the toolbox for modifying the genomes of living organisms, base-editing tools have attracted interest because of specific features: independence of endogenous cellular DNA repair pathways, lower cytotoxic effects than with other Cas9-based tools, high efficacy, which means that there is no need for selection markers, possibility of multitargeting, and the lack of a scar at the edited locus. These properties make BE promising tools for clinical usage in human therapeutics (60) and for multiple applications in plants (51, 61) and microorganisms of medical or biotechnological relevance (62–64). Because of their high efficacy, BE are particularly attractive for bacteria that are difficult to transform, as, ultimately, one single transformant can be enough to obtain a mutant. This is the case for mycoplasmas and other bacteria belonging to the *Mollicutes* class. These minimal bacteria have a limited set of enzymes involved in their DNA repair pathways, and endogenous HR is, for most species, not efficient enough to be used as the basis for “everyday mutagenesis”. In practice, reproducible HR-based mutagenesis with suicide plasmids is only available for Mycoplasma genitalium (65–67), although some mutants were obtained by HR in between chromosome and replicative plasmids for some other species, including Spiroplasma citri (68–70), Mycoplasma pulmonis (71), and M. gallisepticum (22, 72). The low efficiency of HR on the target locus and background integration at the *oriC* (68, 69, 71, 73) makes these tools poorly efficient and therefore not of practical interest. As a consequence, transposon mutagenesis remains the only practical way to generate mutants in many species, including M. gallisepticum (74–77), M. bovis (78, 79), Mycoplasma hyopneumoniae (80), Mycoplasma hyorhinis (81), and many others. While large transposon-based mutant libraries have been obtained to identify essential genes in M. genitalium (82, 83), M. pneumoniae (84), Mycoplasma mycoides subsp*. capri* (85), and Mesoplasma florum (86), the relatively low transformation efficiency of current protocols for some other species, such as Mycoplasma mobile (87) or S. citri (88), is a strong limit for the targeting of a specific gene or group of genes. Because natural recombination events and transformation efficiency are limited for many mycoplasma species, the use of exogenous recombination systems was attempted. Importing the RecA protein from E. coli improved the obtention of bacterial recombinants in Mycoplasma mycoides subsp. *capricolum* (89) and in *M. hyorhinis* (90), but not in M. hyopneumoniae (91). Recently, a RecET-like system from B. subtilis was used for targeted gene inactivation or gene replacement in Mycoplasma pneumoniae (25) and M. gallisepticum (23). However, for now, all of these systems remain limited in efficacy, and their use in a large spectrum of mycoplasmas still needs to be evaluated. In contrast with the situation in eukaryotic cells, CRISPR-Cas9-mediated chromosomal cleavage is often lethal in bacteria, presumably due to the lack of double-strand break repair systems in most of the genera. For this reason, the original S. pyogenes CRISPR-Cas9 system was first used in M. pneumoniae as a counterselection tool, in combination with the exogenous recombination system mentioned above (25). Transcription interference assays using dCas9 (CRISPRi) have been achieved successfully in M. pneumoniae, the synthetic *M. mycoides* subsp. *capri* JCVI-syn1.0 (32), M. gallisepticum and Mycoplasma hominis (92). The use of the mycoplasma endogenous CRISPR-Cas9 system has been described in M. gallisepticum for targeting two genes (93, 94), but unpredictable results have been reported, with mutations localized mostly outside the gRNA targeted sequences for one gene (*ksgA*) and without getting genomic mutants for the second one (*MnuA*). The role of the endogenous Cas9 in these studies remains to be elucidated, and this is due in part to the lack of knowledge about the M. gallisepticum CRISPR-Cas9 system. Further studies are necessary to shed light on the specificity of this system and to explore its possible use as a tool for targeted mutagenesis. In the mycoplasma field, an impactful achievement is the possibility to clone a complete genome in yeast, to modify it in the yeast, and to transplant it back into a recipient cell to generate mutants. Because of the highly efficient HR in yeast, and together with advances in gene synthesis, genetic engineering is now possible at the genome scale using CRISPR-Cas and other genetic tools available in yeast. Currently, this method is available for several species related to the *M. mycoides* cluster (17–19), but it has not yet been extended to members of other phylogenetic groups. In order to circumvent this limit, a recombinase-assisted genomic engineering (RAGE) technology was recently developed and proved efficient to introduce a 15 kbp fragment at a specific locus of the M. pneumoniae genome, and it was able to replace 38 kbp from the genome by means of engineered versions modified either in yeast or in E. coli (59). While they do offer new possibilities of genome engineering in these bacteria, these sophisticated methods are only available for a limited number of species. Therefore, more straightforward and wide-spectrum tools are needed.

In this context, the development of a highly efficient base-editing tool is a significant step forward in the mycoplasma field. Especially, this application of CBE opens new possibilities for targeting multigene families with a limited number of sgRNA molecules. In mycoplasmas, such families are often predicted to encode surface proteins suspected to be involved in host-pathogen interactions, but current mutagenesis methods, including in-yeast genome engineering, are unable to disrupt all of the genes of a family in a reasonable amount of time. In this work, the same promoters were used to drive the expression of the sgRNA (PS) and CBE system (P*xyl/tetO2*) in three species belonging to different phylogenetic groups, suggesting that both expression cassettes could be used without modification in various species of mycoplasmas and possibly other *Mollicutes*. Three different plasmid backbones were used here, including two Tn*4001*-derived transposons and one replicative *oriC* plasmid (Fig. S2). Transposons and *oriC* plasmids are currently the most widely used genetic tools available for *Mollicutes*. Transposon mutagenesis with Tn*4001* derivatives has been used in 15 species, and *oriC* plasmids are available for 14 species. Thus, the mycoplasma CBE system could be easily evaluated in many species, either directly or after cloning the sgRNA and CBE expression cassettes into a compatible vector. During this study, we also found that induction could be performed immediately following plasmid transformation (i.e., immediately after the 2 h cell recovery in Hayflick medium) instead of after colony recovery and regrowth. This improvement allowed us to reduce the duration of the experiments by 14 days and resulted in the expected mutants in less than 3 weeks.

Currently, the base-editing tool described here is limited to C to T mutations. However, the efficacy of base editors is regularly enhanced by combining various enzymes. Adenine deaminase-mediated and dual deaminase-mediated base editing systems have been developed, improved, and validated successfully in various organisms (46, 50, 63, 95, 96). A second limitation comes from the use of SpdCas9 that recognizes canonical NGG-type PAM sites, which limits its target range in genomes. Many variants have now been evolved from SpCas9 to broaden PAM recognition features, including SpCas9-NRRH, SpCas9-NRTH, SpCas9-NRCH (97), SpCas9-NG (98), SpG, and SpRY (99). Cas9 from other bacterial species, with different PAM recognition specificities, including ScCas9 (Streptococcus canis) (100), SaCas9 (Staphylococcus aureus) (101), Nm1Cas9, and Nm2Cas9 (Neisseria meningitidis) (51), have also been used in ABE and CBE systems. Finally, the relatively “wide” editing window of the current CBE might be narrowed by C-terminal truncations of pmCDA1 (102). Therefore, the adaptation of base-editing tools for *Mollicutes*, starting with this work, is open for many further expansions. In particular, the further characterization of endogenous CRISPR-Cas9 systems of mycoplasmas and their PAM requirement may open new opportunities for the design of new base editors and for the expansion of the genetic toolbox for these minimal bacteria.

## MATERIALS AND METHODS

### Oligonucleotides and plasmids.

All oligonucleotides used in this study were supplied by Eurogentec and are described in Table S1. All plasmids constructed and used in this study are listed in Table S2. Detailed protocols for plasmid construction and other methods are provided as SI Materials and Methods.

### Bacterial strains and culture.

M. gallisepticum strain S6 (Tax ID: 1006581) was cultivated at 37°C in modified Hayflick medium (103) in a 5% CO_2_ atmosphere, and puromycin at 10 μg·mL^−1^ and kasugamycin at 400 μg·mL^−1^ were used for selection. M. bovis PG45 (Tax ID: 289397) was cultivated in SP4 medium (103), and gentamicin at 100 μg·mL^−1^ was used for selection. *Mmm* T1/44 (Tax ID: 2103) was cultivated in SP5 medium (18), and puromycin at 8 μg·mL^−1^ was used for selection (104). Phenol red was used as a pH indicator in the mycoplasma media. Escherichia coli NEB-5α (NEB, C2987H) was used for plasmid propagation and was cultivated in Luria broth (ThermoFisher: 12795027), with the addition of ampicillin at 100 μg·mL^−1^ or kanamycin at 50 μg·mL^−1^ for selection.

### Transformation of *Mycoplasma* species.

Mycoplasma transformation was performed using a polyethylene glycol mediated protocol (105, 106). Late log-phase mycoplasma cultures were transformed with plasmid DNA (20 μg). After transformation, cells were resuspended in 1 mL of the appropriate medium, incubated for 2 h at 37°C, and plated onto selective solid medium. After incubation at 37°C for 3 to 10 days, single colonies containing the deaminase constructs were obtained. Expression of the CBE system was induced in early logarithmic growth-phase cultures overnight, and the cultures were plated on selective medium to isolate colonies.
